# Medical and Philosophical Intelligence

**Published:** 1811-02

**Authors:** 


### MEDICAL AND PHILOSOPHICAL INTELLIGENCE.

ROYAL SOCIETY.—The reading of Mr. Davy's Bakerian Lecture was continued on the 6th of December, and concluded on the 13th. In this part of the lecture Mr. Davy detailed a number of experiments, which he regarded as showing that when any metallic oxide is converted into the substance improperly called a muriate, but which is a binary combination of oxymuriatic gas and a metal, the oxygen produced is exactly that which had been absorbed by the metal: and he stated, that the proportions of oxygen or of oxymuriatic gas which combine with metals, are always definite; and that when two proportions combine, the one bears a simple ratio to the other.

Mr. Davy, inferring from the whole series of facts that oxymuriatic gas must be considered as a substance as yet undecompounded, and analogous in many of its properties to oxygen gas, but having stronger attractions for most inflammable bodies, suggests the necessity of altering its name; which conveys so false an idea of its nature.

Conceiving it dangerous in the present improving state of science to adopt any names connected with theoretical arrangements, which may require alteration as knowledge advances, he ventures to suggest for the consideration of chemical philosophers the name of *chlorice*, derived from its green colour; and he proposes to signify its compounds by the name of the basis, with a termination in *ine or ane:* thus hornsilver, improperly called muriate of silver, would be named argent*ane*; muriate of barytes, baryt*ine*, &c.

---

*The Croonian Lecture* on Muscular Motion was read by Mr. Brodie, on the 13th and 20th of December. The subjects introduced in this lecture were less numerous, and the discussions less varied, than usual on similar occasions; and very little or no reference was made to muscular action, the ingenious lecturer confining himself to a simple detail of the thermometrical effects on the animal body, in consequence of dividing the spinal marrow and afterwards inflating the lungs artificially with a pair of bellows, and continuing the circulation of the blood under such circumstances for nearly two hours. The subjects of operation were chiefly rabbits: and the author made a great number of experiments on these animals by dividing the spinal marrow and suffering them to die in this manner, noticing their temperature and that of the room at particular periods; or, after dividing the spinal marrow, inflating the lungs, and thus keeping up the circulation for an hour, and even an hour and a half; noting also the temperature of the heart, intestines, and rectum, at various times during the experiments. The result of the author's inquiries was, that animal heat does not appear to be produced, as generally supposed, by the action of the air on the lungs, and the circulation of the blood; as those animals whose lungs were inflated, and the circulation artificially continued, were always from one to three or four degrees colder in a certain time than those whose spinal marrow was divided and suffered to die. Professor Davy suggested to the author, that the cold air thrown into the lungs (which produced the the usual change in the colour of the blood) might contribute to this effect; and accordingly an experiment was made to obviate such consequence by means of ligature; when it appeared, that in an hour and forty minutes the body in which the circulation was artificially continued after dividing the spinal marrow, was only one degree colder than that which died immediately. Mr. B.'s experiments seem to militate against the doctrine of the vitality of the blood; but they do little towards illustrating the fact, that tortoises can live and walk about long after having been deprived entirely of the brain, and even part of the spinal marrow.

On the evening of the 20th, part of a letter from Dr. Parry, of Bath, was read, on certain nervous affections; as convulsions, tremulous motions, and sudden startings or pulsations of what is vulgarly called the life blood.

---

Royal Society of Edinburgh.—On Monday the 5th of November, the Royal Society of Edinburgh met for the first time in their new apartments in George-street, when Dr. Thomas Thomson read two papers, giving the account of the analyses of two new minerals from Greenland. To one of them he has given the name of allanite, and to the other sodalite. In the first he discovered a considerable portion of cerium, and in one analysis he detected a quantity of a metallic oxide perfectly new in its properties, for which he proposed the name of junonium. The other mineral, according to his investigation, affords 23 per cent. of soda and three of muriatic acid. By an analysis of Mr. Ekeberg, the same constituents were yielded in the proportions of 25 per cent. and six per cent.

At the next meeting on the 19th, a short communication was read, respecting a singular water-spout observed at Ramsgate.

On the 3d inst. a paper by Dr. Brewster was read, being a new demonstration of the fundamental properties of the lever.—Also a communication by Sir George Mackenzie, bart. relative to the hot springs of Iceland; when Sir George exhibited some beautiful drawings and part of a series of magnificent specimens from that country, which he proposes to deposit in the cabinet of the Society: and at the last meeting, on the 17th, Sir George began a description of the minerals of Iceland, when he exhibited specimens from the district called the Guldbringe Syssel.

---

*Vaccination in France.* The following document, which appears to be official, points out the system pursued by the French legislature to promulgate vaccination, and to disseminate the evidence of the benefit arising from adopting that discovery.

Ten years of labour and success have at length decided the important question, as to the vaccine possessing the power of preserving all those in whom it has regularly gone through its progress, from the small-pox. This has been carried to such a degree of certainty by the experiments of the Central Committee, and its numerous correspondents, as well Frenchmen as strangers, that there is not at present any fact in medicine better proved, or more certain, than that which establishes the truly *anti-variolous* power of the Vaccine.

His

His Majessty the Emperor and King, to whom the different reports of the Central Committee have been presented, has been sensible of the advantages which would result from the general propagation of the new inoculation. His Majesty has seen the preservation and increase of the population of his vast empire, immediately connected with the adoption of this method. He has had an account rendered to him of the obstacles which, in some districts, might yet oppose its progress, and has found that these consisted principally in the great difficulty of procuring and preserving the vaccine fluid.

In consequence, his Majesty, wishing to give to his people a signal mark of his paternal solicitude, has granted to his Excellency, the Minister of the Interior, an annual and special credit, destined to provide for the expences necessary for extending the practice. He has formed, in twenty-four of the principal cities of France, depots of the vaccine fluid, where every one who wishes to practice vaccination may be sure of always finding disposable matter. These are, Besançon, Bourdeaux, Brussels, Caen, Clement-Ferrand, Dijon, Florence, Lisle, Lyons, Marseilles, Mentz, Montpellier, Nancy, Nantes, Orleans, Parma, Rheims, Rennes, Rouen, Saintes, Strasbourg, Thoulouse, Tours, and Turin.

His Majesty has further created a Vaccine Committee at each of these depots, and has preserved to the Central Committee, established near his Excellency the Minister of the Interior, its original organization, in charging it with the central depot of Paris.

In conclusion, his Majesty has appointed, by his decree of the 6th of November last, annual rewards for those who shall have performed the greatest number of operations—collected the most important facts—overcome most obstacles—and arrested the course of variolous epidemics. These rewards have been so distributed that every effort has been noticed, and every labour proportionably recompensed.

They are thus determined:—1st, A prize of 3000 francs; 2d, two prizes of 2000 francs; 3d, Three prizes of 1000 francs; and 4th, a hundred silver medals bearing the head of the Emperor.

These powerful motives always to keep up and preserve the Vaccine fluid; this energetic incentive to an emulation which must direct all the efforts of the practitioners towards a rapid propagation of the Vaccine, leads to the hope that the public communication of his Majesty's benevolent intentions, will be sufficient to give a general impulse in favour of the new method, and banish in a few years that scourge, the small-pox, from the French territory.

Already the returns of the mortality in the city of Paris, for the year 1809, exhibit only 213 deaths, by small-pox. This number, though yet too considerable, since the vaccine offered to these 213 victims a certain method of preservation, is yet extremely small in comparison of that of some years, when the epidemic small-pox has carried off, in the same city, more than 2[illegible],000 individuals. The Committee does not hesitate to attribute this diminution of mortality to the zeal with which the different Members, who compose it, have extended the practice in the large establishments to which they are attached, as physicians and surgeons; to the efforts of all the professional men, and of some ecclesiastics in the capital; and lastly to the conspicuous exertions of Messieurs the Counsellors of State, the Prefects of the Seine and of Police, the the Mayors and their assistants, who have always seconded the Committee with the greatest zeal, and in many instances anticipated its intentions.

Every good man, and every friend to mankind, may hope then, that the new measures taken by his Majesty will at length effect that which the labours of the Committee have long led them to expect. There is reason to believe, that they will so stimulate the emulation of all physicians and surgeons, that very shortly the small-pox, already unknown in many departments, where the zeal of the prefects has been such that there remain none to vaccinate but the infants born in every year, will entirely disappear from France, as the leprosy has done, of which no traces are found, except in the history of the worst governed ages of our Monarchy.

The Committee embraces this opportunity of reminding the public, that the Central Establishment of the Vaccine, founded the 7th of February, 1801, and situated in No. 1, Rue du Battoir, St. Andre-des-Arcs, is still carried on; that vaccination is practised gratuitously there every Tuesday and Saturday, at noon; that the children of the poor are admitted gratuitously during the course of the Vaccine; and that application for vaccine fluid should be addressed, under cover to his Excellency, to M. Husson, Physician of the Hotel Dieu, and of the Imperial Lyceum, Secretary to the Committee.

Given at the sitting of the 11th of May, 1810, the day of the 10th Anniversary of the foundation of the Committee.

Signed by all the Members.—Duchanoy, *President;* Corvisart, Delasteyrie, Doussin-Dubreuil, Guillotin, Hall, Hazard, Jadelot, J. J. Leroux, Marin, Mongenot, Parfait, Pinel, Salmade, Thouret—Husson, *Secretary.*

---

*Inquiries concerning the Eau Medicinale and the Rhododendron Chrysanthum.*

Has the Rhododendron Chrysanthum been lately used in Scotland? Has it obtained any reputation for the cure of gout or rheumatism? Is it cultivated in Britain? If not cultivated in this country, where or how is the genuine plant to be procured? Have the other species of rhododendron any medical powers?

The reasons for wishing to obtain information on these points, are the following: A medicine has lately been imported from France under the title of Eau Medicinale d'Husson, and now much used for the relief of the paroxysms of gout, with great efficacy and perfect safety. The effects and operation of the Eau Medicinale, and those said to arise from the use of rhododendron, are so strikingly similar, that it is suspected the Eau Medicinale is prepared from the rhododendron. The Eau Medicinale is professed to be prepared from a vegetable substance, which vegetable is said to be discovered, and an imitation of the Eau Medicinale is made from it, and advertised for sale in London, but still as a secret.

Dr. Home's experiments with rhododendron were unsuccessful in acute rheumatism, but it was found to be active and powerful, and as it has obtained a place in the Materia Medica of the Edinburgh Pharmacopœia, I am in hopes, that you, Gentlemen, or some of your readers, will will be able from later experience, to say whether it is possessed of the virtues attributed to it by Koelpen, aud others upon the Continent.

In Dr. Woodville's Medical Botany, there is a figure of the Rhododendron Chrysanthum. It is a native of Siberia, and he says is not yet introduced into Britain. I have not found it in the catalogues of our nurserymen, nor in our plantations and shrubberies; but my local and confined situation may be the means of my not knowing where it is to be procured.

Should my suspicions prove well-founded, and the rhododendron be possessed of similar powers to the Eau Medicinale, it will be an acceptable service to gouty sufferers, and also to the faculty, to rescue an useful medicine from the hands of Quackery.

The Editors of the Edin. Jour. to whom these inquiries were addressed, say, that the last Rhododendron Chrysanthum used in Scotland, was a parcel of the dried leaves sent from Russia by Dr. Guthrie of St. Petersburgh, to Dr. Duncan, senior. It was introduced into the Edinburgh Pharmacopœia, chiefly on account of the reputation it had procured in Russia as a cure for rheumatism; but the trials here did not correspond with its reputation, as we never saw any remarkable benefit from its use. In particular, it never produced a purgative effect, and is therefore probably not the basis of the *Eau Medicinale*, which is said to operate powerfully even in small doses. It has not yet been cultivated in Scotland, although the rhododendron Dauricum has been mistaken for it. It is however mentioned in Don's catalogue of the Cambridge Botanical Garden, as introduced there in 1801, and is, as might have been supposed, marked *hardy*. It is figured in the Botanical Magazine for March or April last. The Rhododendron Ferrugineum is supposed to possess similar virtues.

We have lately been informed, that the digitalis lutea is supposed to be the basis of the *Eau Medicinale*. *Edin. Jour.* 1811.

---

*Extract of a Letter concerning the Empirical Gout Medicine, called Eau Medicinale d'Husson.*

A number of respectable testimonies, it must be granted, have appeared within the last six or eight months in favour of this nostrum. But a keen and experienced observer will not feel conviction, notwithstanding the tide of popular opinion: 1. Because he will recollect, that on former occasions the public admitted the testimonies of patients, and even of medical men, as quite satitfactory of the efficacy of various gout medicines, which, however, proved to enjoy but a temporary existence.

2. Because the influence of public opinion is at present such as to carry efficacy along with the exhibition of any medicine which is not positively hurtful, but especially if it be a medicine which is really beneficial on account of purgative, and perhaps some sedative virtues.

3. The shocking ignorance of medical science displayed in the printed directions by the author of the Eau Medicinale d'Husson, at least proves that the public is not indebted to any person capable of adequate observation, for this medicine.

4. The known discredit, and even prohibition of this nostrum in Paris, serve to shew, that in all probability it has, on trial, been proved not to answer the expectations of the Parisians.

With

With respect to the composition of the present favourite gout medicine, conjectures only are offered. It seems to contain, in a vinous solution, a purgative ingredient in a very small quantity of matter; a slightly bitter ingredient; and perhaps a narcotic or sedative one. The foxglove was early suspected as one of the articles, and although many trials seem to have disproved its existence in this preparation, some persons still are of opinion that it is one of the ingredients. Tobacco has been proposed, but on no just gronnd whatever. Toadstool has been named, on account of some similarity of colour and smell, and exhilarating effects. The purgative ingredient has been suspected by some to be scammony, by others to be elaterium.

I have just had a bottle of the Eau Medicinale shewn to me which has been twenty-five years in London; it was administered at that time but gained no credit.

It is not intended by these observations to discourage prudent trials of the medicine in question, for certainly the evidence in its favour is quite enough to justify them; but, as it is of importance that the truth be determined as soon as possible, medical practitioners may probably be assisted by the above observations.

---

### *Case of Rupture of the Gall-Bladder.*

An athletic man, about forty years of age, after violent and protracted sufferings from a remittent fever, was for many weeks afflicted with symptoms of derangement of the stomach and chylopoietic viscera. He had a fixed pain at the pit of the stomach, constant nausea, and a short time after eating, the food was generally rejected in the form of chyme. The bowels were very torpid, and his stools untinged with bile: at length he became jaundiced, and for many weeks suffered all the aggravated symptoms of that disease in a violent degree. One morning, whilst raising himself in bed, he was suddenly seized with a most violent pain in the right hypochondrium, which in a very few minutes extended throughout the whole abdomen. The pain rapidly increased; the belly almost immediately became tense and much swollen; the pulse was quick, contracted, and intermittent; his respiration was difficult; his extremities cold; his countenance ghastly, and he had constant vomiting. These symptoms increased for forty-eight hours, when the abdomen was as much swoln as in an advanced state of ascites. The pain and tension were excessive: he became delirious and died. During this time fomentations were applied to the abdomen; he was repeatedly bled, and anodyne glysters were injected, with very little and but temporary relief. Upon dissection, upwards of three gallons of yellow serum mixed with flakes of lymph were first drawn off. The peritoneum lining the muscles and covering the anterior surface of the intestines was universally inflamed, and of as deep a scarlet colour as the conjunctiva in the most acute stage of opthalmia. The bowels were collected in the posterior part of the cavity, and glued together by a layer of coagulated lymph, which adhesion bounded the inflammation, for it did not in the least degree extend between their convolutions, although extreme on their anterior surface. They were much distended with air, and the pelvis was filled with maases of lymph. The edge of the right lobe of the liver adhered firmly to the beginning of the arch of the colon; and between the concave surface of the liver and the arch of the colon a large cyst was formed, which had burst by an ulcerated aperture, about as large as a sixpence, into the cavity of the abdomen. When pressed externally, a quantity of thick bile issued from this aperture: when cut into, it was found to be of sufficient size to hold a quart of fluid, and contained the gall bladder, which communicated with the cyst by an apparently ulcerated opening of considerable size, a little above its fundus.

Thus the gall bladder had first given way, and the issued bile had caused this *épanchemen* between the liver and colon, which had ulcerated from distention, and the effusion of its contents into the cavity of the abdomen had produced this fatal inflammation.

The pancreas formed a tumor as large as an orange, a section of which exhibited the membranous bands peculiar to the schirrous structure: the pressure had obliterated the ductus communis choledicus, and communicated by a ragged ulcerated surface with the cavity of the duodenum. The liver and other viscera were natural.—*Lond. Med. Rev.*

---

The Spring Courses of Lectures at St. Thomas's and Guy's Hospitals, will commence the 1st of February, viz. *at St. Thomas's*, Anatomy, and the Operations of Surgery, by Mr. Cline, and Mr. Astley Cooper. Principles and Practice of Surgery by Mr. Astley Cooper. *At Guy's*, Practice of Medicine, by Dr. Babington, and Dr. Curry.—Chemistry, by Dr. Babington, Dr. Marcet, and Mr. Allen.—Experimental Philosophy, by Mr. Allen.—Theory of Medicine, and Materia Medica, by Dr. Curry, and Dr. Cholmeley.—Midwifery, and Diseases of Women and Children, by Dr. Haighton.—Physiology, or Laws of the Animal Œconomy, by Dr. Haighton.—Structure and Diseases of the Teeth, by Mr. Fox.

---

Dr. Watt, of Glasgow, will begin his summer course of Lectures on the Theory and Practice of Medicine, on Monday the 6th of May.

---

Dr. Hooper, and Dr. Ager, will re-commence their Lectures on the Theory and Practice of Physic, Chemistry, and the Materia Medica, at the Theatre in Cork-street, Burlington Gardens, on Monday, February 4, 1811, at Eight o'clock in the Morning.

---

Dr. Millar, of the University of Glasgow, has in the press, Disquisitions on the History of Medicine, exhibiting a view of Physic, as observed to exist during remote periods, and among nations not far advanced in refinement.

---

Mr. White has nearly ready for the press, the Flora of the counties of Northumberland and Durham, of which the Botanist's Guide through those counties may be considered as the Prodromus. It will comprize about two thousand indigenous plants, and be illustrated trated by some coloured engravings, from drawings made by Sowerby.

---

Sir George Alley, M. D. of Fermoy, Ireland, is engaged in arranging for the press, a work to be entitled "Reports of the Utility and Employment of Mercury, in the Treatment of Inflammatory, and other diseases, in which the exhibition of that remedy has been neglected, or considered as inadmissible." As this publication will necessarily embrace many important medical subjcts, the author earnestly solicits the assistance of the respectable practitioners, civil and military, of Great Britain and Ireland, and will be happy to acknowledge any communications with which he may be favoured.

